# Laser-Assisted Visible-Light Polymerization for Rapid Synthesis of Molecularly Imprinted Polymers

**DOI:** 10.3390/bios15080529

**Published:** 2025-08-13

**Authors:** Wissal Mrabet, Abdelhafid Karrat, Aziz Amine

**Affiliations:** Laboratory of Process Engineering and Environment, Faculty of Sciences and Techniques, Hassan II University of Casablanca, P.A. 146, Mohammedia 28806, Morocco; wissal.mrabet@etu.fstm.ac.ma (W.M.); abdelhafid.karrat@etu.fstm.ac.ma (A.K.)

**Keywords:** MIP, laser-assisted polymerization, visible light photoinitiation, bisphenol A, sulfamethoxazole, erythrosine B

## Abstract

The demand for rapid, energy-efficient, and low-toxicity methods for synthesizing molecularly imprinted polymers (MIPs) is increasing, particularly for applications in environmental monitoring and green chemistry. In this context, the present work focuses on the development of a novel laser-assisted method for MIP synthesis, employing a visible laser (450 nm) and erythrosine B as a green photoinitiator. This visible-light approach enables fast and spatially controlled polymerization while avoiding the drawbacks of conventional methods (thermal heating, UV synthesis), such as the use of toxic initiators like AIBN and the need for UV shielding. MIPs were synthesized for bisphenol A and sulfamethoxazole, two emerging contaminants of significant environmental concern. The synthesis process was optimized for rapidity and scalability, and the resulting MIPs were integrated into a paper-based analytical device (MIP-PAD) for smartphone-assisted, on-site detection. The developed sensors exhibited excellent analytical performance, with recovery rates of 98.6% in tap water and 90.2% in river water and relative standard deviations (RSDs) below 1.88%. This study demonstrated a green, efficient, and highly controllable laser-assisted polymerization technique, offering a promising alternative to conventional MIP synthesis methods.

## 1. Introduction

MIPs are specifically designed synthetic materials that possess recognition sites, enabling them to selectively rebind a target molecule even in the presence of closely related compounds [1,2,3]. These materials are created by polymerizing functional monomers around a template molecule, resulting in a crosslinked three-dimensional network polymer. The monomers are selected based on their ability to interact effectively with the functional groups of the template molecules. After polymerization, the template molecules are removed, leaving binding cavities in the polymer network that are complementary to the target compound in terms of size, shape, and functionality [4]. The resulting imprinted polymers exhibit stability, robustness, and resistance to a broad range of temperatures, solvents, and pH [5].

MIPs are widely applied in both sensing and non-sensing fields due to their good selectivity and stability. In non-sensing applications, they are used for the controlled release of active compounds, solid-phase extraction, and selective purification processes [6,7]. In sensing applications, MIPs are integrated into gas and liquid-phase sensors for various analytical purposes [7,8]. They have been successfully employed in the detection of pharmaceuticals and drugs of abuse, environmental pollutants and pesticides, food contaminants, explosives, pathogens, chiral compounds, and biomarkers for medical diagnostics [9,10,11,12,13,14]. Additionally, MIPs show great promise in emerging areas such as packaging contamination detection, air quality monitoring, pharmaceutical quality control, and the development of smart drug delivery systems [15,16,17]. MIPs are becoming increasingly popular alternatives to natural receptors in fields such as food safety, security, health, and environmental monitoring. This is because they are low-cost to produce, chemically stable, and can be used with many types of sensors, such as electrochemical, optical, and plasmonic systems [18,19,20,21].

MIPs are mainly synthesized using techniques such as bulk polymerization, suspension polymerization, precipitation, and emulsion polymerization. In addition, energy-assisted methods like microwave polymerization and ultrasound-assisted synthesis are also employed, depending on the intended application [3,4]. However, they have some limitations that may prevent their wide application, most notably the requirement for a long polymerization time and exact reaction conditions to achieve reproducibility. In this study, we addressed the challenge of rapid polymerization, which is a major obstacle in MIP synthesis. Using laser-assisted polymerization methods, we explored a promising approach that enables fast initiation of the polymerization process, which considerably reduces the overall synthesis time of MIPs for SMX (sulfonamide antibiotic) and BPA (an industrial chemical commonly found in plastics); both are emerging contaminants frequently detected in environmental water sources. As is known, photopolymerization requires a photoinitiator to initiate the polymerization reaction. In this work, we used erythrosine B (ErB) as a photoinitiator because it offers several advantages: it is a readily available, cost-effective, and water-soluble dye that exhibits strong adsorption in the visible light range. Indeed, ErB has previously been employed as a photoinitiator in polymer synthesis [22,23]. However, to the best of our knowledge, this is the first time ErB has been used for the development of a molecularly imprinted polymer (MIP) via photopolymerization. Additionally, its low toxicity and compatibility with aqueous systems make it a favorable choice for environmentally friendly and biocompatible MIP synthesis. One of the key advantages of using visible lasers lies in their ability to avoid high-energy UV radiation, which can degrade sensitive template molecules or functional monomers; they also offer better penetration depth, minimize side reactions, and offer significantly higher safety in handling compared to UV radiation, making the process more controllable, user-friendly, and suitable for scale-up.

## 2. Materials and Methods

### 2.1. Reagents

The following products were employed in the synthesis of MIPs: SMX and BPA as the template molecules, 99% pure methacrylic acid (MAA) as the functional monomer, ErB as the photoinitiator, ammonium persulfate (APS), at 98%, as the co-photoinitiator, and 98% ethylene glycol dimethacrylate (EGDMA) as the cross-linker. All of them were obtained from Sigma-Aldrich, St. Louis, MO, USA. Dimethyl sulfoxide (DMSO) of ≥99% purity was provided by Loba Chemie, Mumbai, Maharashtra, India.

Methanol (≥99.9%), purchased from Loba Chemie, was used for the template removal process (Mumbai, Maharashtra, India). Acetic acid (≥99%) was purchased from Honeywell Fluka (Wunstorfer Strasse, Seeltze, Germany).

For the colorimetric detection of SMX and BPA, sodium nitrite (NaNO_2_, 99%), N-(1-naphthyl) ethylenediamine dihydrochloride (NED), and sulfamic acid were purchased from Merck (Darmstadt, Germany), while hydrochloric acid (HCl) and sodium nitrite were also obtained from Sigma-Aldrich (St. Louis, MO, USA).

To evaluate the performance of the MIP-PAD under real environmental conditions, water samples were collected from the Oued Malh River in Casablanca, Morocco.

### 2.2. Instrumentation and Image Processing Method

The synthesis of MIPs was carried out with a CNC machine (VEVOR 3018 Pro, Birmingham, UK) equipped with a UV laser diode module (Oxlasers, Shenzhen, China) operating at a wavelength of 450 nm and an output power of 5 W, which was also employed for the photopolymerization process. Glass microfiber filters with a diameter of 47 mm and a pore size of 1.2 µm were acquired from FiltraTECH (Saint Jean de Braye, France) and served to support the MIP. A Samsung Galaxy M13 smartphone was used to capture an image and measure the RGB color intensity using the “RGB color” application. Fourier transform infrared (FT-IR) spectroscopy analysis was performed using a Frontier Perkin-Elmer spectrometer (PerkinElmer, Waltham, MA, USA) capturing spectra from 4000 to 500 cm^−1^ to verify the chemical structure and functional groups of the developed MIPs.

### 2.3. Procedure for the Colorimetric and Electrochemical Determination of SMX and BPA

The colorimetric detection of SMX was performed by adding successively 20 µL of 1% sodium nitrite (NaNO_2_), 20 μL of hydrochloric acid (HCl, 1 M), 20 μL of sulfamic acid (2% *w*/*v*), and 20 μL of N-(1-naphthyl) ethylenediamine dihydrochloride (NED, 1% *w*/*v*) to the MIP. Concerning BPA detection, the procedure included mixing 50 μL of sulfamethoxazole (4 mg/mL), 50 μL of hydrochloric acid (2 M), and 50 μL of sodium nitrite (23 mg/mL). Subsequently, the mixture was added to the MIP containing PBA, and the addition of 100 μL of phosphate buffer (0.5 M, pH = 12) resulted in a visible yellow color, confirming the presence of BPA [22]. For smartphone detection, the pink and yellow colors developed by SMX and BPA, respectively, were captured by A Samsung Galaxy M13 smartphone. The images were processed by a color picker application to measure the RGB color intensity channels (red, green and blue). After comparing the color intensities, the channel most sensitive to the analyte concentration was used for SMX and BPA analysis. It should be noted that in image analysis, RGB intensity reflects the individual values of red, green, or blue color channels. Strong visible colors often result from low intensity in the opposite channel. For example, a strong pink color appears as a low-intensity green. This creates an apparent inverse relationship between detected RGB intensity and the visible color. All measurements were performed in triplicate.

For the electrochemical detection of SMX, 100 mg of MIP was first incubated with a solution containing a known concentration of SMX under agitation for 10 min. After this binding step, the supernatant was removed. Then, 0.1 M phosphate-buffered saline (PBS) at pH 7.0 was added to the MIP to facilitate the release of the bound SMX. This desorption step was carried out for 10 min to ensure complete release. The released SMX was then detected using a screen-printed electrode (SPE) with the square wave voltammetry (SWV) technique, using a frequency of 10 Hz, an amplitude of 20 mV, and a step potential of 5 mV.

### 2.4. Synthesis of MIPs Using Laser-Assisted Visible-Light Polymerization

**Scheme 1:**
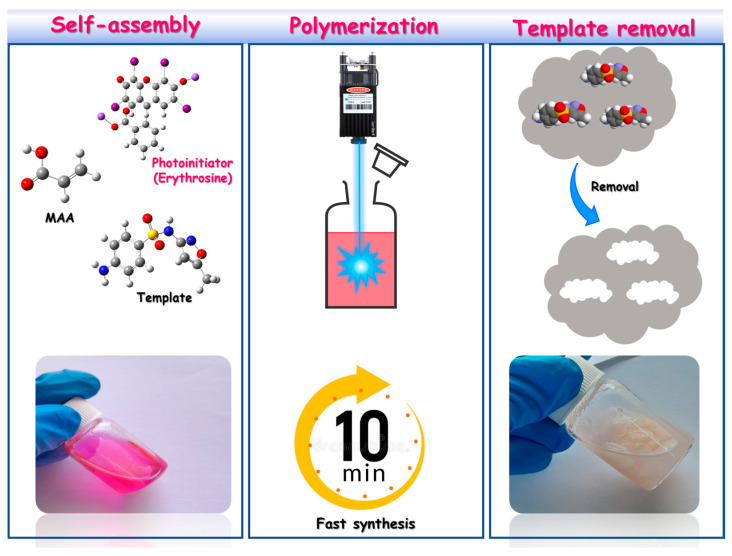
Schematic illustration of MIP synthesis using laser-assisted synthesis.

The MIP preparation shown in Scheme 1 was performed by dissolving 5 mg of SMX in 2.5 mL of DMSO. The solution was then incubated for 1 h:30 min with 175 µL of MAA, allowing for the formation of non-covalent hydrogen bond interactions between the monomer and the template. Following the self-assembly step, 2.5 mL of water was added, followed by 320 µL of EGDMA as a cross-linker (0.3 M), 0.09 mM ErB as a photoinitiator, and 17 mM of APS as coinitiator.

Polymerization was carried out under exposure to a laser diode (450 nm wavelength) for 10 min. The same procedure was applied for the preparation of an MIP designed for the detection of BPA, using 3 mg of BPA as a template instead of SMX. The template was removed using a methanol and acetic acid mixture (9:1, *V*:*V*) for 1 h and 30 min. Additionally, a non-imprinted polymer (NIP) was synthesized using the same procedure as the MIP, but without the addition of the template.

### 2.5. Preparation of MIP-Integrated PAD

**Scheme 2:**
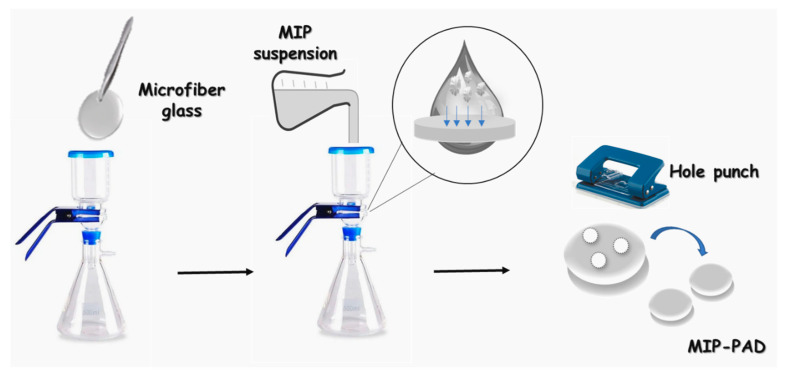
The procedure for the preparation of an MIP integrated into PAD.

The preparation of the MIP-integrated PAD is shown in Scheme 2. The first step includes adding 30 mg of MIP into 5 mL of distilled water into a tube compatible with a vacuum filtration system. After applying vacuum pressure, the MIP suspension is immobilized onto the glass fiber, resulting in an MIP-integrated paper. Finally, small disks are punched out using a hole puncher.

### 2.6. Selectivity and Stability Studies

To evaluate the selectivity of the developed MIP-PAD, structurally similar molecules including SND, SDZ, SCT, and SMR were tested at a concentration of 20 ppm following the same detection procedure described above. The color intensity of each interferent was measured by a smartphone. Moreover, a stability test was also conducted. Briefly, the developed MIP-PAD (without the SMX template) was stored at 50 °C for 26 days. Measurements were performed on days 1, 12, and 26. On each measurement day, an adsorption test was carried out: a fixed concentration of SMX (20 ppm) was added to the PAD.

### 2.7. Determination of SMX in Water Samples

Water samples, including tap and river water, were spiked with 5 ppm of the target analyte. Then, 60 µL of each sample was added to the MIP-PAD following the same detection procedure described above. The final concentration was used to calculate the recovery and relative standard deviation (RSD) of the MIP-PAD sensor. All measurements were performed in triplicate.

## 3. Results and Discussion

### 3.1. FTIR Characterization

**Figure 1:**
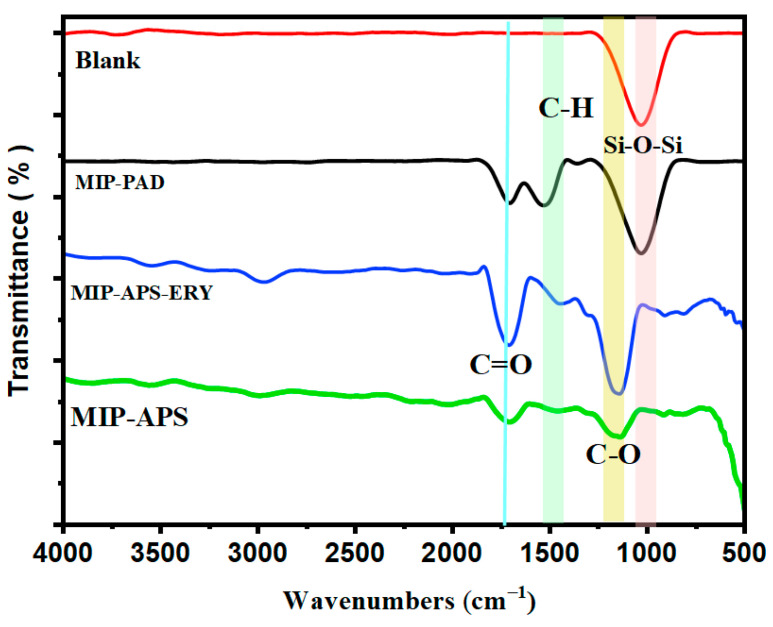
FTIR spectra of PAD, MIP-PAD, MIP-APS-ErB, and MIP-APS.

FTIR spectroscopy was used to characterize the chemical functional groups of the MIP-integrated PAD (Figure 1). The PAD spectrum showed a characteristic stretching band around 1000 cm^−1^, confirming the presence of Si–O–Si. In the case of MIP–PAD, additional bands appeared at 1720 cm^−1^ and 1500 cm^−1^, corresponding, respectively, to C=O stretching and C–H bending vibrations (from MAA and EGDMA) [23], while the Si–O–Si band at 1000 cm^−1^, typical of silica, remained visible. This indicates the presence of both functional groups from the functional polymer and the silica matrix. In the spectrum of MIP–APS, bands were observed at 1720 cm^−1^ (C=O) and 1500 cm^−1^ (C–H), along with a peak at 1150 cm^−1^ attributed to C–O stretching, indicating also the presence of functional groups of polymer MAA [24]. The MIP–APS–ErB complex exhibited the same characteristic peaks as MIP–APS, confirming that ErB did not modify the chemical composition of the polymer [25].

### 3.2. Comparison Between Synthesis Techniques

The synthesis of the polymer was carried out using several methods, as outlined in Table 1, each offering distinct advantages and limitations in terms of time and efficiency. Among these, the laser-assisted method stands out for its remarkably short processing time, which is only 10 min, three times faster than the ultrasound bath technique. In contrast, the conventional heating method at 60 °C requires approximately 3 h to complete, making it less suitable for applications where rapid production is essential. Moreover, the laser synthesis method does not require high temperatures or prolonged energy input, unlike heating or ultrasound-based methods, making it a more energy-efficient method. This low energy requirement, combined with the absence of high thermal stress, makes the laser method especially suitable for synthesizing polymers intended for use with temperature-sensitive analytes such as proteins. Given these advantages, the laser synthesis method demonstrates strong potential for time-efficient, energy-saving, and high-quality polymers, particularly in bioanalytical and environmental applications. Moreover, the proposed synthesis method was compared with previously reported methods, which typically require long synthesis times ranging from 8 to 24 h. In contrast, the present method significantly reduces both time and energy consumption

### 3.3. Selection of the Initiator

In this study, a laser-induced polymerization technique was employed using a 450 nm laser source to initiate polymerization in the presence of various initiators (Table 2). ErB, a xanthene-based dye, was found to successfully initiate polymerization under visible light irradiation, albeit slowly, with polymer formation occurring after 1 h. This behavior is due to ErB’s ability to absorb in the visible region (maximum absorbance around 520–540 nm) with sufficient absorbance at 450 nm to allow excitation under the applied laser. Upon photoexcitation, ErB transitions to its singlet- and subsequently triplet-excited state, which can interact with dissolved oxygen or initiate polymerization directly through radical formation. However, when APS or AIBN were used alone, no polymerization was observed. This is expected, as both are thermal initiators that do not absorb visible light and therefore cannot generate radicals under irradiation at 450 nm. Notably, the combination of ErB with APS led to a marked acceleration of the polymerization process, with gel formation occurring within just 10 min. This enhanced effect can be attributed to a photoinduced electron transfer mechanism, where the excited ErB^∗^ molecule donates an electron to the persulfate anion (S_2_O_8_^2−^), resulting in the formation of sulfate radical anions (SO_4_•^−^), which are highly reactive initiators of free radical polymerization. The key initiation reaction can be written as follows:

ErB^∗^ + S_2_O_8_^2−^     →    ErB^•+^ + SO_4_^•−^ + SO_4_^2−^

The generated sulfate radical (SO_4_•^−^) initiates polymerization by attacking the vinyl group of the monomer, forming monomer radicals that propagate the polymer chain. This represents a new example of a photoinitiation system, where the dye acts as a photosensitizer and the persulfate functions as the electron acceptor and radical precursor. In contrast, the mixture of ErB and AIBN did not result in polymerization under 450 nm light, as AIBN does not participate in photochemical electron transfer and requires thermal decomposition (typically at ~60–80 °C) to generate radicals. These findings demonstrate that ErB can act as a visible-light photoinitiator and that its performance can be significantly enhanced via a photo-redox mechanism in the presence of APS, making this combination highly effective for visible-light-driven radical polymerization.

### 3.4. Choice of the Color Channel for the Detection of SMX and BPA

**Figure 2:**
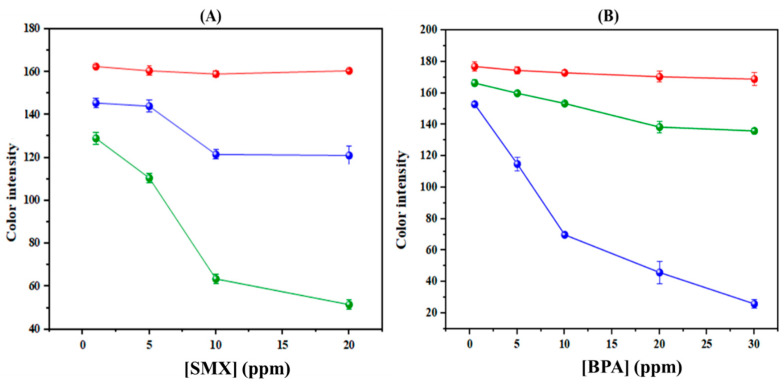
Correlation analysis of red, green and blue channels for (**A**) SMX concentration varying from 1 to 20 ppm; And for (**B**) BPA concentration varying from 0.5 ppm to 40 ppm.

Figure 2A illustrates the correlation analysis of blue and green measurements for concentration of SMX ranging from 1 to 20 ppm, indicating the green signal had the strongest correlation with the pink color, confirming that the green color is the most efficient for the detection of the SMX. Meanwhile, the results for BPA in Figure 2B show that the blue channel is the most optimal choice for quantifying BPA.

### 3.5. MIP Performance

**Figure 3:**
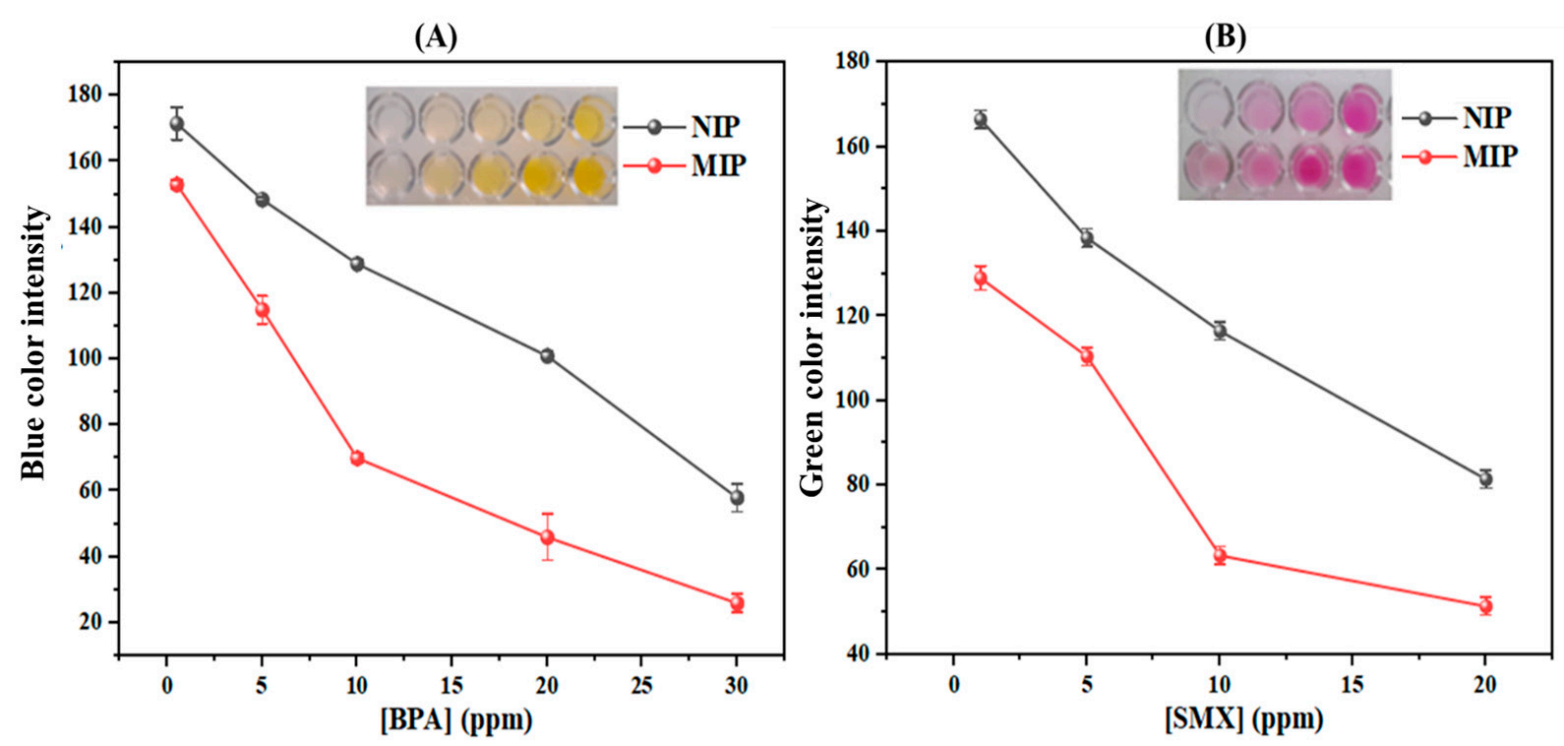
(**A**) Evaluation of the binding affinity of BPA on MIP and NIP by blue color intensity changes. (**B**) Evaluation of the binding affinity of SMX on MIP and NIP by green color intensity changes.

The performance of the MIP cavities was evaluated across a concentration range of SMX (1–20 ppm). As shown in Figure 3B, the green color intensity decreases progressively with increasing SMX concentration. This decrease is more pronounced for the MIP compared to the NIP, indicating a higher adsorption capacity and stronger affinity of the MIP toward the SMX template. These results confirm the successful formation of specific recognition cavities within the MIP structure.

Similarly, in the case of BPA detection, the MIP also shows a notable decrease in blue color intensity with increasing BPA concentration, indicating efficient binding due to the presence of specific recognition sites in the polymer matrix. The NIP, on the other hand, exhibits only a slight variation in color intensity, confirming its lower affinity for BPA in the absence of these specific binding sites. Moreover, ErB was successfully used to initiate the polymerization of the MIP matrix, resulting in the formation of well-defined cavities that match the target molecules.

### 3.6. MIP Integrated into PAD

**Figure 4:**
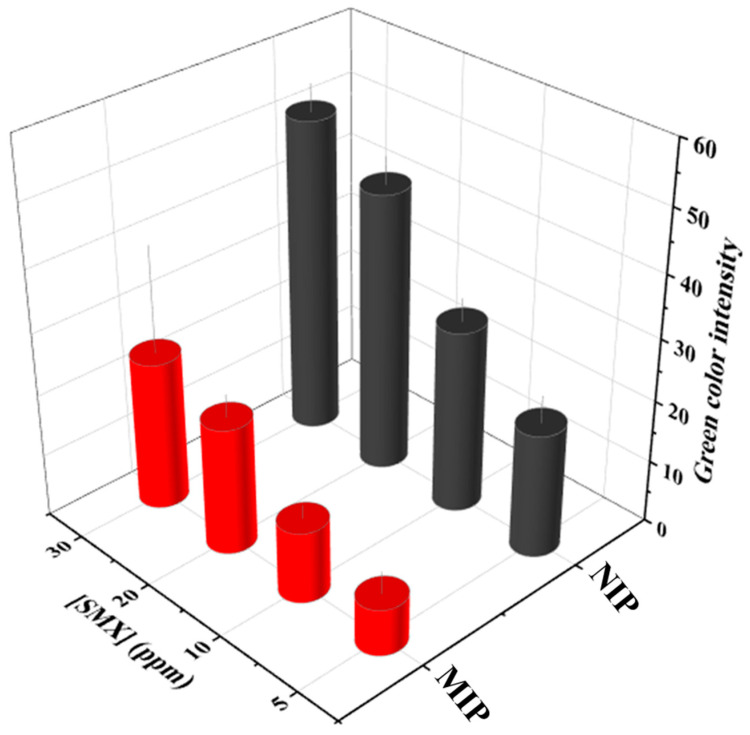
Calibration curves based on green intensity examining the binding affinity on MIP-PAD and NIP-PAD.

As shown in Figure 4, the PAD integrated with both the MIP and NIP exhibited changes in green color intensity upon exposure to increasing concentrations of SMX. A pronounced increase in color intensity was observed for the MIP at all tested concentrations, clearly surpassing the response of the NIP. This enhanced response highlights the superior recognition capability of the MIP, attributed to the specific binding sites (cavities) created during the molecular imprinting process, which enable selective interaction with SMX. In contrast, the NIP presented a weak and less responsive signal, consistent with nonspecific adsorption. Notably, the MIP synthesized using the laser-assisted polymerization technique was successfully integrated into the PAD, forming an MIP-PAD platform for colorimetric detection. This approach offers several advantages, including low cost, portability, ease of fabrication, and the potential for on-site, real-time monitoring without the need for complex instrumentation. These findings confirm both the efficiency of the laser-assisted MIP synthesis and the practical applicability of the MIP-PAD for selective, in situ detection of SMX in environmental and pharmaceutical monitoring [26].

### 3.7. Electrochemical Detection of SMX

**Figure 5:**
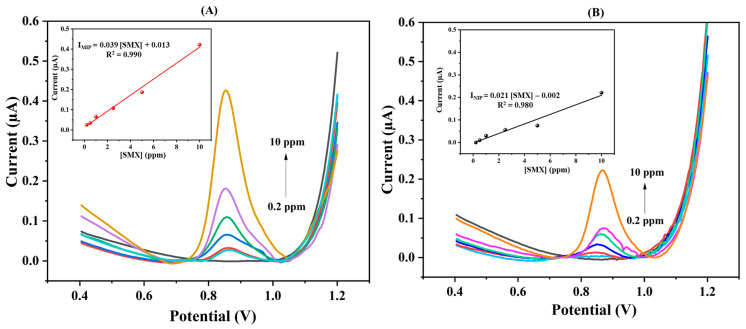
SWV of the SMX using MIP (**A**) and NIP (**B**). Parameters: frequency of 10 Hz, amplitude of 20 mV, and step potential of 5 mV.

To confirm the versatility of the developed MIP synthesized via laser-assisted polymerization, we applied it for the electrochemical detection of SMX. Figure 5 demonstrates that the MIP produced a stronger current response than the NIP across the entire concentration range. This suggests that the MIP can better recognize and capture the target molecule (SMX) due to the specific binding sites. Using electrochemical detection with MIP significantly enhances the sensor’s sensitivity. It allows for the detection of very low concentrations, even below 1 ppm, with reliable results at levels as low as 0.2 ppm. This makes the developed MIP sensors highly selective and sensitive for detecting SMX by both colorimetric and electrochemical detection.

### 3.8. Selectivity Study

**Figure 6:**
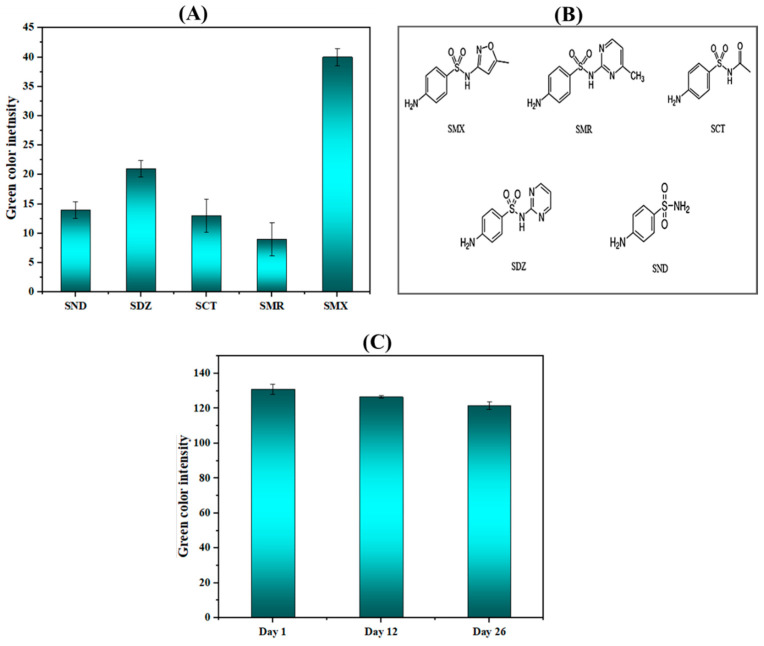
(**A**) Selectivity study of the developed MIP-PAD (concentration of 20 ppm). (**B**) Structure of similar SMX analogues used for the selectivity study. (**C**) Stability study of MIP-PAD.

The selectivity evaluation of the synthesized MIP-PAD highlighted its ability to specifically recognize SMX among various structurally similar sulfonamides. As shown in Figure 6, the green color intensity, which reflects the adsorption level, is significantly higher for SMX compared to the other sulfonamides, such as SND, SDZ, SCT, and SMR. This pronounced difference confirmed the presence of highly specific recognition sites within the polymer matrix, formed through the molecular imprinting process.

During the laser assisted-synthesis, SMX was used as the template molecule, enabling the creation of binding cavities that are complementary in size, shape, and functional groups. The strong affinity of the MIP-PAD for SMX indicates successful imprinting. This ability to discriminate between closely related molecules makes the MIP-PAD a promising tool for environmental monitoring, chemical analysis, and targeted SMX detection with minimal interference from similar compounds [30].

### 3.9. Stability Study

To evaluate the stability of the MIP for SMX detection, a 26-day study was conducted under storage conditions at 50 °C, with regular monitoring of the green color intensity, which indicates the sensor’s detection capability. As illustrated in Figure 6C, the MIP maintained strong performance during the first 12 days, with only a slight decrease in signal observed compared to day 1. By Day 26, a more noticeable, yet still moderate, reduction in intensity was recorded. This gradual decline suggests that the MIP remains effective for at least two weeks under elevated temperature conditions and retains acceptable functionality even after nearly a month. These results highlight the good thermal stability of the MIP, making it a reliable option for SMX detection in demanding environmental settings.

### 3.10. Real Sample

To evaluate the applicability of the MIP-based sensor in real sample analysis, recovery experiments were carried out using tap water and river water samples spiked with 5 ppm of sulfamethoxazole (SMX). The results in Table 3 demonstrate satisfactory recovery rates of 98.6% for tap water and 90.2% for river water, indicating the method’s good accuracy and minimal matrix effect. The relative standard deviation (RSD) values of 1.88% and 1.54%, respectively, further confirm the method’s precision and reproducibility. These findings validate the potential of the developed MIP sensor for the reliable detection of SMX in complex environmental water samples.

## 4. Conclusions

In this study, a rapid and effective method for synthesizing MIPs was developed using laser-assisted photopolymerization in the visible light range. By using ErB as a photoinitiator and APS as a coinitiator, we successfully demonstrated a significant reduction in polymerization time from several hours to 10 min without impacting the performance of the MIP, including specificity, selectivity, and stability. The developed MIP exhibited high selectivity and sensitivity for the detection of SMX and BPA, as confirmed through both the colorimetric and electrochemical methods. The successful integration into a PAD highlights their potential for portable, low-cost, and efficient performance.

Overall, this work presents a robust, eco-friendly, and scalable approach to MIP fabrication using visible lasers, thereby opening up new opportunities for the development of advanced sensors in environmental, pharmaceutical, and food safety applications.

## Figures and Tables

**Table 1:** Comparison of the present synthesis method with previously reported methods.

Synthesis Method	Monomer	Initiator/Photointiator	Time	Ref
Radical thermal polymerization in solution (water bath)	MAA	AIBN	8 h	[26]
Thermal polymerization	MAA	AIBN	24 h	[27]
Radical thermal polymerization in bath (oil bath)	MAA	AIBN	20 h	[28]
Thermal polymerization in bath (oil bath)	MAA	AIBN	24 h	[29]
Laser polymerization	MAA	ErB	10 min	This work
Ultrasound bath	MAA	ErB	30 min	This work
Thermal heating	MAA	ErB	3 h	This work

**Table 2:** Effect of different initiators on polymerization efficiency under 450 nm laser irradiation.

Initiator	Polymerization	Time	Comment
ErB	Yes	1 h	ErB absorbs visible light at ~500–550 nm and partially at 450 nm. Upon photoexcitation, it can generate radicals directly or through interaction with oxygen.
APS	No	—	APS is a thermal initiator; it does not absorb visible light, so it cannot generate radicals under 450 nm irradiation.
ErB + APS	Yes	10 min	ErB photoexcites under 450 nm light and transfers electrons to APS, accelerating radical generation via a photo-redox mechanism.
AIBN	No	—	AIBN is a thermal initiator and requires heating (~60–80 °C) to decompose into radicals; it is not light-sensitive.
AIBN + ErB	No	—	No synergistic effect; AIBN does not participate in photochemical electron transfer with ErB under visible light.

**Table 3:** Data obtained from analyzing tap and river water samples for detecting SMX using MIP-PAD.

Water	Added (ppm)	Found (ppm)	Recovery	RSD (*n* = 3)
Tap	5 ppm	4.93	98.6%	1.88%
River	5 ppm	4.51	90.2%	1.54%

## Data Availability

The data used to support the findings of this study are available from the corresponding author upon request.
